# Hyperbranched polyester amide/polyethersulphone mixed matrix nanofiltration membranes for contaminant rejection

**DOI:** 10.1039/d4ra08400d

**Published:** 2025-01-21

**Authors:** Ayman El-Gendi, Mona H. Abdel Rehim

**Affiliations:** a Giza Engineering Institute Giza Egypt; b Chemical Engineering Department, Engineering Research Institute, National Research Centre Giza Egypt; c Packing and Packaging Materials Department, Institute of Chemical Industries Research, National Research Centre 33 El Behooth St., Dokki Giza Egypt monaabdelrehim23@gmail.com mh.abdelrehim@nrc.sci.eg +20 2 33371718

## Abstract

Nanofiltration (NF) separation technology is a low-pressure filtration process, which is highly efficient and environmentally friendly. As a result, it has found wide application in water treatment. This work describes the preparation of flat sheet membranes *via* the phase inversion method using blends of hyperbranched polyester amide (PEA) and polyether sulphone (PES) in definite ratios. The obtained mixed matrix membranes were characterized using FTIR, TGA and contact angle analysis, and their morphologies were investigated using SEM. SEM images showed a porous membrane with micro-voids found underneath, confirming the suitability of the membranes for nanofiltration. Adding PEA to PES changed the porosity, which changed the membrane performance. Examining the removal of heavy metals [Pb(NO_3_) and CuSO_4_] using the prepared membranes revealed that the NF membranes had a higher salt rejection efficiency than pure PES with a good permeate flux. M3 membrane showed 81% rejection of Pb (NO_3_)_2_, while M2, the membrane with a low PEA ratio, rejected 85%, with high water flux for both membranes. Moreover, the presence of PEA in the membrane tissue led to protein rejection up to 99.5%. Thus, these novel blend membranes proved themselves as NF-type membranes with better performance in water treatment.

## Abstract 2

Nanofiltration (NF) separation technology is a low-pressure filtration process, which is highly efficient and environmentally friendly.

## Graphical abstract

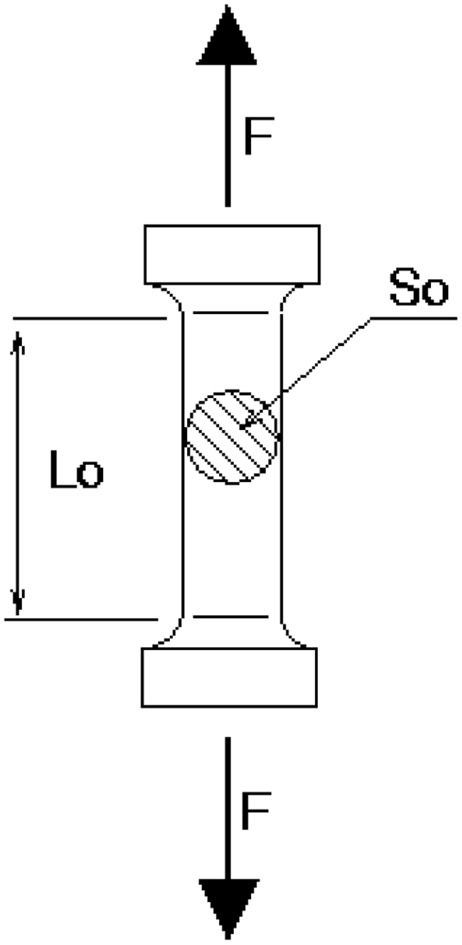

## 1 Introduction

Water treatment is performed for removing microorganisms and natural and man-made chemicals from water to enhance its quality. The quality of the water supply determines the methods used for water treatment. However, to deactivate any pathogenic bacteria that are present in the water, it must always be disinfected. This method has so far been shown to be the most crucial for preserving human life. Chlorine or chlorine dioxide is frequently utilized for disinfection. Nevertheless, other methods, including ozonation and UV irradiation, are also frequently employed. Moreover, other techniques are used to remove chemicals such as coagulation/flocculation, sedimentation, and filtration. However, membrane processes like nanofiltration (NF) and ultrafiltration (UF) have gained increasing popularity in water treatment.^1^ Nanofiltration is a pressure-driven technology that drives the passage of monovalent ions through small pores and traps divalent and hypervalent ions.^2^ Nanofiltration (NF) uses membranes with very small pores (∼1 nm) and requires operating pressures below 10 bar. NF has some advantages over other membrane methods, such as high rejection of divalent ions, low operating pressure, high flux, and low energy consumption. These features make NF a promising and innovative technology that can be widely applied in the treatment of drinking water and industrial effluents.^3,4^

Polyethersulphone (PES) is a common polymer used for NF membrane fabrication. The membrane is prepared *via* a phase inversion method in which an exchange between a solvent in the polymer solution and its non-solvent occurs in a coagulation bath, yielding a porous membrane. However, the membranes formed from PES are of high chemical and thermal resistance since they are composed of rigid phenyl rings connected with SO_2_ groups. These hydrophobic membranes suffer from rapid membrane fouling and flux decline. Moreover, cleaning PES membranes with oxidizing agents such as chlorine and hydrogen peroxide causes membrane degradation. To increase membrane hydrophilicity and enhance its permeability, blending of PES with polymers, copolymers or inorganic materials is reported in literature.^5–9^ Synthesis of NF *via* blending PES and sulfonated polysulfone yielded a loose NF membrane, which was able to selectively remove pharmaceutical and personal care products (PPCPs) from drinking water.^10^ A new technique to fabricate a NF membrane for wastewater treatment based on modified graphene oxide (GO) with triethylenetetramine (TETA), CuFe_2_O_4_, and acetic acid (AC) (supported GO-TETA-CuFe_2_O_4_@AC) as a supported protic ionic liquid (PIL) modifier for polyethersulfone was described by Gholami *et al.*^11^ The modified NF membrane showed high reflux and good removal ability compared to the unmodified membrane. Moreover, the newly prepared NF membrane exhibited high salt rejection up to 97% for BaCl_2_ and obvious chlorine resistance. A polyethersulfone (PES) NF membrane incorporated with graphene oxide (GO) functionalized with 3-aminopropyltriethoxysilane (APTS) was prepared by a non-solvent phase inversion method.^12^ This modification step resulted in increased rejection of protein bovine serum albumin (BSA), sunset yellow and acridine orange dyes, and divalent salts (MgSO_4_).

Hyperbranched polymers (HBPs) are a class of macromolecules that received great interest in the last few decades because of their unique structures and properties. They are distinguished by their branched chains and large number of functional end groups.^13^ These two features enabled HBPs to be a suitable candidate for many applications such as catalysis, drug delivery, biomimetics, and coatings among others.^14–18^ HBPs are easy to synthesize from multifunctional monomers, making them more favorable in industrial applications. However, attempts have been made to add HBPs as additives in porous membranes.^19–21^ Zhao *et al.* reported the use of hyperbranched polyglycerol (HPG) as an additive to prepare a polyvinylidene fluoride (PVDF) membrane *via* the phase inversion process.^22^ The results showed that higher contents of HPG in the casting solution led to an increase in surface pore size and porosity of the membranes, which in turn increased the pure water flux of the blend membranes. Zhao *et al.*, in another study, studied the effect of the arm length of amphiphilic hyperbranched-star polymers on the hydrophilicity and protein resistance of PVDF membranes.^23^ It was reported that increasing the arm length of the star polymer led to the enrichment of the membrane surfaces with the star polymer substantially. Consequently, this results in a significant decrease in water contact angle, lower static protein adsorption, higher protein solution fluxes, and better protein solution flux recovery. Preparation and characterization of NF membranes synthesized by using hyperbranched polyester and terephthaloyl chloride (TPC) were reported.^24^ The flux and rejection of these NF membranes for Na_2_SO_4_ (1 g L^−1^) reached 11.43 L m^−2^ h^−1^ and 96.5%, respectively, under 0.6 MPa. Han *et al.* synthesized composite membranes from hyperbranched polyamide amine modified by palmitoyl chloride and polyether sulphone using the phase inversion method.^25^ The formed membranes showed an efficiency of 86% in Cd(ii) ion removal from wastewater, which highlights the potential application of these composite membranes in wastewater treatment.

A hollow fiber NF membrane with a modified active thin film composite (TFC) was reported.^26,27^ The modification was carried out through the incorporation of cross-linked hyperbranched polyester. The formed membranes demonstrated high dye removal and salt rejection with good flux and long-term separation capacity. Recently, Kanjorian *et al.* described the fabrication of a PPES NF membrane modified by hyperbranched polyamidoamine functionalized with cucurbituril for heavy metal rejection and dye removal.^28^ The obtained modified membrane has a smooth surface with high hydrophilicity, which enabled it to have high flux, and highest flux with a permeability of 8.5 L m^−2^ h^−1^ bar^−1^. Moreover, ionic hyperbranched poly(amido-amine) was incorporated in NF membranes for desalination and dye removal.^29^ The presence of ionic HBPs facilitated ionic dye/salt selectivity by endowing the nanochannels with charge characteristics. The performance of new membranes based on hyperbranched polymers is being investigated in this work. Using the phase inversion method, a combination of PEA and PES is employed to create a membrane. It is investigated how the shape, porosity, and membrane performance are affected when hyperbranched polyester amide is added to the membrane matrix. Additionally, the membranes' capacity to eliminate heavy metals and protein adsorption is examined.

## 2 Materials and methods

### 2.1 Materials

Phthalic anhydride (Ph-An), diisopropanol amine (DIPA), and *n*-hexane were purchased from Fluka. Polyether sulphone (*M*_w_ = 94 000 g mol^−1^) and polyvinyl pyrrolidone (PVP, *M*_w_ = 35 000 g mol^−1^) were supplied by BASF. *N*-Methyl-2-pyrrolidone (NMP) was purchased from Merck. Novatexx 2483 nonwoven was provided by Freudenberg Filtration Technologies SE & Co. KG. PVP was dried for 16 h at 60 °C in a vacuum and stored airtight. Disodium hydrogen orthophosphate anhydrous and monosodium dihydrogen orthophosphate heptahydrate were procured from CDH Chemicals Ltd, India. Bovine serum albumin (BSA), *M*_w_ = 69 kDa, was purchased from Sigma-Aldrich and was used for preparing protein feed solution. All chemicals were used as received without further purification. A commercial PES membrane supplied by NADIR Filtration GmbH (Wiesbaden, Germany) was utilized for comparison.

### 2.2 Methods

#### 2.2.1 Synthesis of PEA

PEA was prepared by the method reported in the literature^30,31^ with some modification, as follows: Phthalic anhydride (0.17 moles) is reacted with D1PA (0.23 moles) under a nitrogen atmosphere. The temperature was raised to 140 °C for 3 hours. After complete dissolution of anhydride, a vacuum was applied to remove H_2_O for 2 hours. The reaction was stopped when the reaction mixture turned into a yellowish viscous liquid. The prepared polymer is soluble in water and methanol, besides highly polar solvents. The obtained polymer has ester and amide groups in the backbone and OH end groups as confirmed by FTIR (yield = 87% (45 g)).

#### 2.2.2 Thermal treatment of PEA

PEA was heated for 2 h under air at 170 °C in a thermal oven, then collected and reweighed. The resulting treated polymer is insoluble in water but still soluble in DMSO.

#### 2.2.3 Membrane preparation

**Scheme 1:**
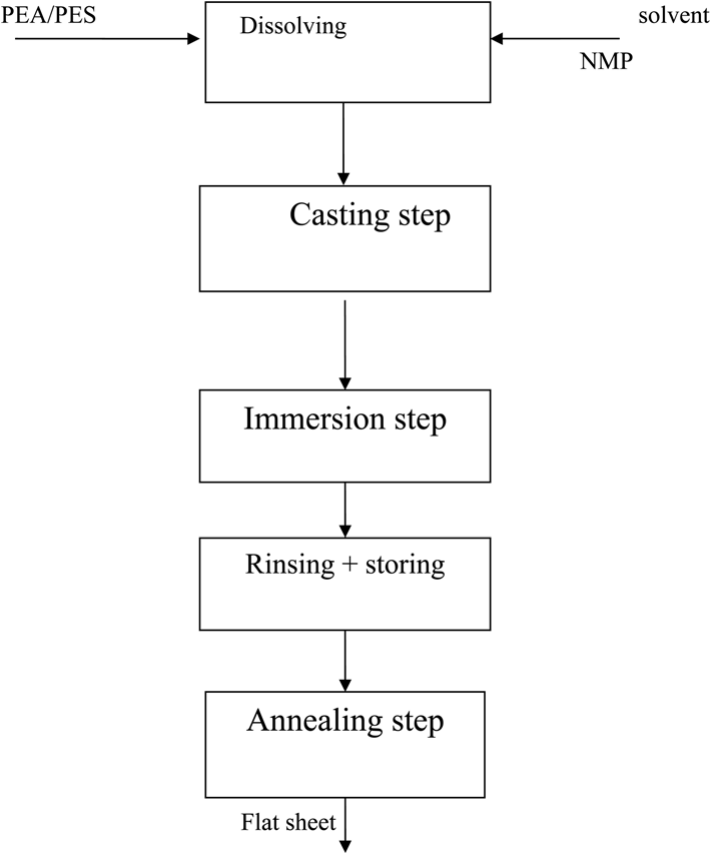
Process flow sheet for membrane preparation.

The hyperbranched polyester amide (PEA) polyethersulphone (PES) membranes were prepared by the phase separation method. The blend solutions based on synthesized PEA and PES polymers were prepared according to Table 1 by dissolving the two polymers at different compositions in NMP as the solvent (Scheme 1). The nonwoven sheet was pasted to a glass substrate and soaked for a few minutes in PVP/H_2_O solution (40 : 60 v/v). The homogeneous blend solution was degassed under vacuum and cast on the nonwoven sheet using a blade casting knife of 100 μm thickness. The cast membranes were moved to the coagulation bath for immersion precipitation and kept in the bath for 15 min. In the final stage, the membranes were rinsed with deionized water carefully, and stored in a water bath at 4 °C. Before testing, the membranes were annealed for 1 hour at 100 or 120 °C (near *T*_g_) in a vacuum oven. Pure PES membranes were prepared in the same manner based on the similarity in the complex viscosity of the blend membranes. The pure membranes are denoted PES15 to be compared with M2, and PES18 to be compared with M3.

**Table 1:** Amounts of PEA, PES, and NMP used to prepare the asymmetric membranes

Membrane	PEA (wt%)	PES (wt%)	NMP (wt%)
M1	12.5	16	71.5
M2	14.25	14.25	71.5
M3	17.5	11	71.5
PES 15	—	15	85
PES 18	—	18	82

### 2.3 Membrane characterization

#### 2.3.1 Fourier transform infrared spectrometer (FT-IR)

A Fourier transform infrared spectrometer (FTIR-6100) from Jasco Inc. was used to investigate the chemical structure of the prepared membranes.

#### 2.3.2 Nuclear magnetic resonance spectroscopy

^1^H NMR-measurements were carried out on a Bruker Avance III spectrometer (Bruker Biospin, Germany) at 500.13 MHz (^1^H), and solvent signal served as the internal chemical shift reference for ^1^H (DMSO-d_6_: 2.51 ppm).

#### 2.3.3 Size exclusion chromatography

The molecular weight of HP-PEA was determined using a Knauer GPC equipped with a refractive index detector (K2301) and a light scattering detector MiniDAWN LS (Wyatt Technology). The sample was dissolved (2 mg mL^−1^) in DMAc containing 3 g L^−1^ LiCl and the same solvent served as the eluent. Measurements were conducted at a flow rate of 1.0 mL min^−1^ at room temperature. A Polar Gel-M column (300 mm × 7.5 mm, Agilent Technologies) was used.

#### 2.3.4 Scanning electron microscopy (SEM)

Scanning electron microscopy (SEM) was used to inspect the cross-section and surface of membranes. The surface morphology was examined using a JEOL-HRSEM scanning electron microscope (SEM). The samples were fractured in liquid nitrogen for cross-section view and all samples were sputtered with gold before imaging with SEM.

#### 2.3.5 Thermo-gravimetric analysis (TGA)

Thermo-gravimetric analysis (TGA) was conducted with a SETARAM Setsys TG-12 System at a heating rate of 10 °C min^−1^. The thermal stability and the loss of 10 wt% of the PEA/PES membranes as a function of temperature were evaluated using a magnetic microbalance of high accuracy (2 × 10^−6^ mg). The PEA/PES samples were cut from the prepared membranes and dried under vacuum. The TGA measurements were carried out in a nitrogen atmosphere from 30 to 1000 °C at a heating rate of 10 °C min^−1^.

#### 2.3.6 Mechanical property

**Fig. 1:**
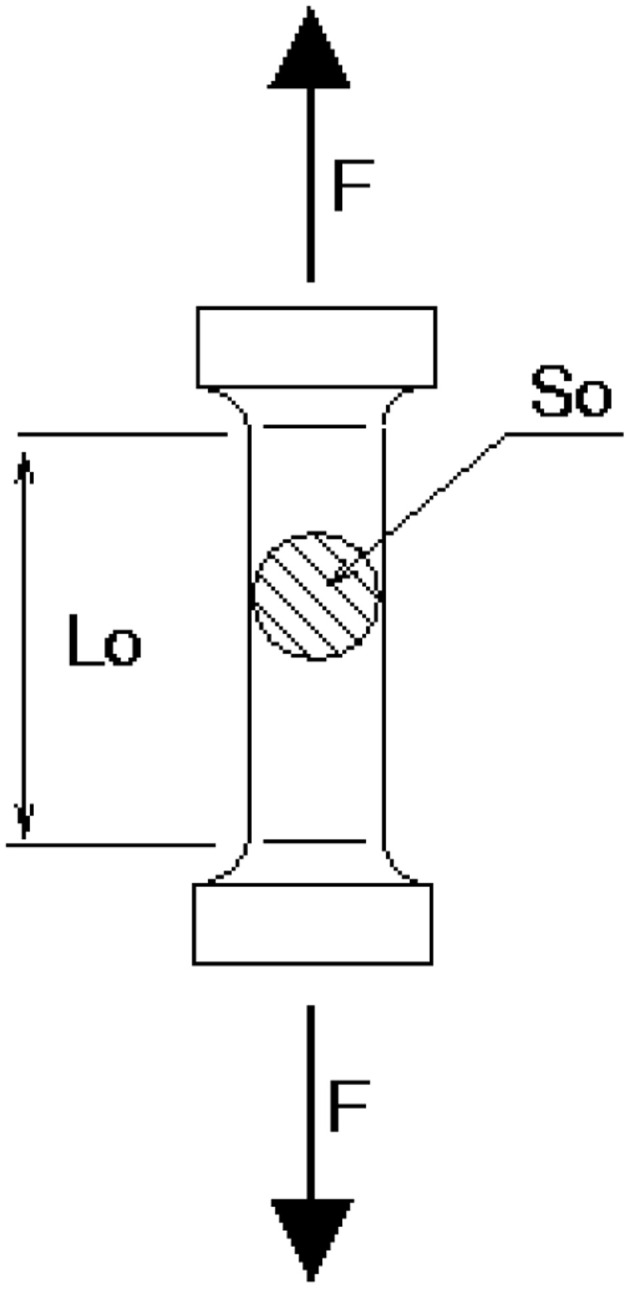
Shape of PEA/PES specimens used in mechanical testing.

The tensile strength of PEA/PES membranes was measured by using an Instron Tensile Tester 5569 (universal tensile testing machine). The mechanical properties of the prepared PEA/PES were determined using test specimens as shown in Fig. 1. The test specimens were prepared by the standard method. These specimens are cut from PEA/PES films. Young's modulus, tensile stress and elongation at break reported are the average of several measurements.

#### 2.3.7 Water uptake

The water uptake property is an important characteristic of a flat sheet membrane since it is directly related to the hydrophilicity and porosity of the membrane skin.^32^ To test the maximum water content durability, the PEA/PES membranes were immersed in clean water for 24 hours. The percent of water content (WC) was calculated using the equation

1 WC% = [*W*_wet_ − *W*_dry_/*W*_dry_] × 100

where *W*_wet_ and *W*_dry_ are the wet and dry weights of the membranes, respectively.

### 2.4 Membrane performance

**Fig. 2:**
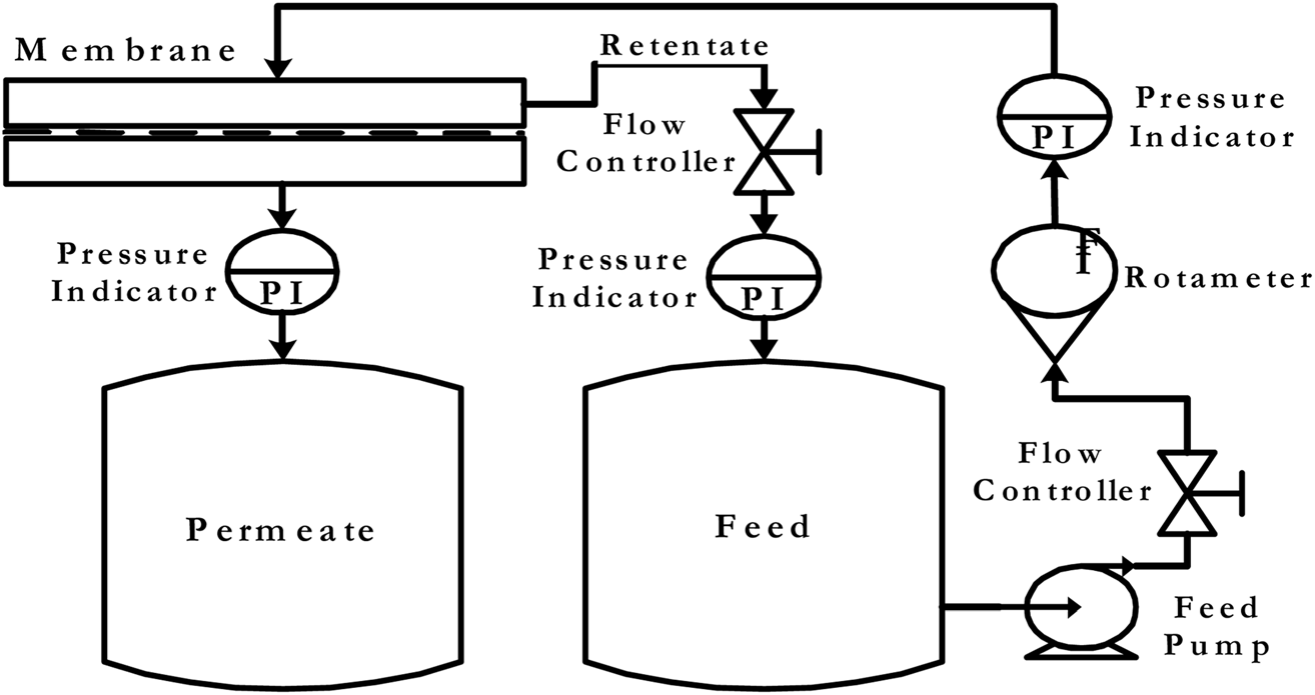
Instrument set-up for membrane testing.

The membrane performance as a function of different operating parameters has been investigated using the membrane testing set-up instrument described in Fig. 2. The prepared membranes are examined for the removal of heavy metals such as Pb(NO_3_)_2_ and CuSO_4_. The concentration of Pb(NO_3_)_2_ solution is 200 ppm, while for the CuSO_4_ solution two different concentrations are tested, which are 200 and 400 ppm. A saline solution of concentration 200 ppm is pumped at a pressure of 4 bar through the prepared membrane. After that, the permeate degree of salinity was estimated to test the membrane efficiency. The effective membrane surface area is 19.6 cm^2^. The total flux (*J*) of the tested solution was determined using the following equation:

2 *J* = *Q*/ (*A* × *T*_*t*_)

where *Q* is the permeate mass in kg, *A* is the membrane active area in m^2^ and *T*_*t*_ is time in hours.

The membrane permeability is calculated based on flux = *J*/pressure while the salt rejection (*R*) was calculated as

3 *R*(%) = (1 − *C*_p_/*C*_f_) × 100%,

where *C*_f_ is the concentration of the feed solution and *C*_p_ is the concentration of the permeate. *C*_p_ and *C*_f_ were measured with a conductivity meter.

### 2.5 Protein–bovine serum albumin (BSA) adsorption

Protein adsorption tests were conducted by using bovine serum albumin (BSA) of concentration 1 g L^−1^ as the feed solution. 100 mL of phosphate buffer saline (PBS) consisting of 137 mmol L^−1^ NaCl, 2.7 mmol L^−1^ KCl, 10 mmol L^−1^ Na_2_HPO_4_·12H_2_O, and 2 mmol L^−1^ KH_2_PO_4_ was added to the feed solution before testing. The filtration test was conducted in three stages; the first stage was membrane compaction through pumping DI water as feed for 3 hours until a steady state of membrane flux was achieved. The second stage was the filtration of the protein solution. The third stage was cleaning the membrane with DI water for 3 hours until constant permeate flux was obtained. The protein concentration in the permeate solution was evaluated by using RotiQuant® protein assay.^33^ The absorbance of protein solutions at 595 nm was used to determine the protein concentration. Protein concentration was calibrated with protein solutions of known concentration in the range from 5 to 50 mg L^−1^. The amount of adsorbed protein *A*_BSA_ is calculated using eqn (4) and is given in percentage of rest protein concentration *c*_R_ in the solution to the initial concentration *c*_0_.4
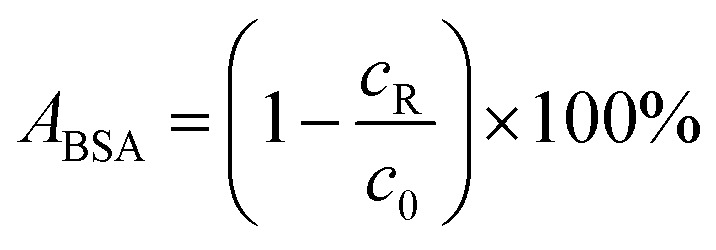


## 3 Results and discussion

### 3.1 Hyperbranched polyester-amide (PEA)

**Scheme 2:**
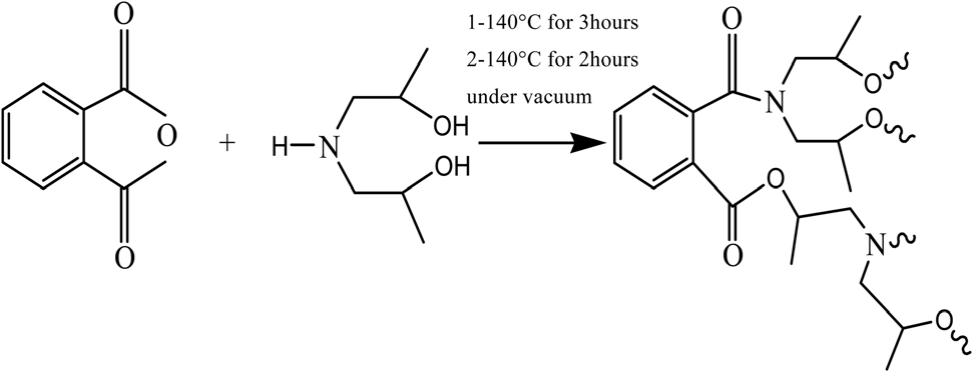
Synthesis of PEA *via* polycondensation reaction.

Hyperbranched polyester-amide was prepared from commercially available monomers. The detailed polymer synthesis was described elsewhere^30^ but the used method is modified by changing the molar masses of the reacting monomers and temperature scheme. The condensation reaction of the two monomers yielded an *in situ* intermediate, which under the reaction's conditions undergoes further polycondensation reaction to yield hyperbranched polyester-amide (Scheme 2). The formed polymer is soluble in water and methanol and its chemical structure contains amide and ester groups and a large number of hydroxyl end groups. The chemical structure of PEA was confirmed by ^1^H NMR using DMSO as the solvent. The following peaks were assigned: (DMSO-d_6_) *δ* ppm = 7.3–8.1 aromatic protons; 4.8–5.1 (OH); 4.17 (CH_2_–OCO); 3.9 (CH_2_–N); 3.73 (CH̲_2_OH); 3.52 (N–CH̲_2_–CH_2_–OCO); 3.5 (N–CH̲_2_–CH_2_–OH); 1.2 (CH_3_). The formed hyperbranched polymer is water soluble so it was important to inhibit its water solubility through thermal treatment of the polymer by heating it under vacuum for two hours at 170 °C. This process led to a polymer dissolving only in highly polar solvents such as DMF, DMSO and NMP. The thermal treatment process did not affect the chemical structure of the hyperbranched polymer as confirmed by ^1^H NMR. It is assumed that partial cross-linking occurred between the polymer chains, which reduced water solubility. The molar mass of the polymer was measured using gel permeation chromatography (GPC) and was found to be 43 000 g mol^−1^ after the thermal treatment. However, there is no noticeable change in the polymer molar mass between before and after the thermal treatment, which indicates that no degradation in the polymer backbone has occurred.

### 3.2 Membrane fabrication

The membrane development by the phase inversion method is governed by kinetic and thermodynamic parameters. Exchange between the solvent and non-solvent, polymer–solvent interaction, solvent–non-solvent interaction and interfacial stability are among these parameters.^34^ Therefore, the selection of the solvent, non-solvent, and polymer material is very crucial in asymmetric membrane preparation. NMP was selected as the solvent in the membrane fabrication process since it is a strong polar polymer and widely used in the phase inversion method. Variation of polymer to solvent ratios is widely used for membrane preparation for different applications.^35–37^ In our approach, the ratio of the polymer to solvent is maintained constant. Nevertheless, the ratio of PEA to PES in the blend is changed to study the effect of the presence of the HBP on the membrane tissue. The cast membranes were annealed for 1 hour at 100 or 120 °C in order to study the effect of annealing temperature on membrane performance and rejection.

### 3.3 Membrane characterization

#### 3.3.1 Infrared spectra (FT-IR)

**Fig. 3:**
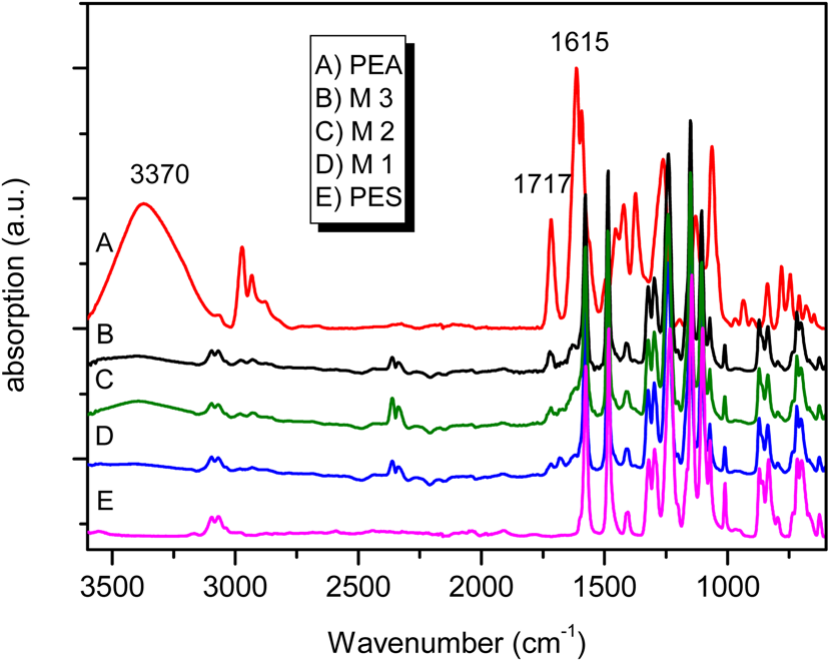
FTIR spectra of different PEA/PES blends.

To follow the proper course of the PEA/PES synthetic steps, FT-IR was used. The IR spectra obtained for M1, M2 and M3 membranes are presented in Fig. 3 besides the spectra of pure PEA and PES. These spectra reveal the characteristic bands expected for PEA/PES blends such as 3370 cm^−1^ (OH bonded), 2964 cm^−1^ (*ν*_as_, CH_2_) and 2840 cm^−1^ (*ν*_s_, CH_2_), 1717 cm^−1^ (C=O, ester linkage) and 1615 cm^−1^ (N–C=O, amide I). These features indicate that despite the thermal treatment the characteristic groups of PEA are present. Moreover, the characteristic bands of polysulphone are overlapped by those of HB–PEA and it is difficult to differentiate between the bands belonging to each polymer.

#### 3.3.2 Differential scanning calorimetry (DSC)

The compatibility of the two polymers was examined by DSC and measuring the *T*_g_ of the obtained membrane. The *T*_g_ values for pure PES and PEA are 234.1 and 87.9 °C, respectively. Blending of PEA with PES and increasing the amount of PEA have led to a decrease in *T*_g_ value as can be noticed from the values presented in Table 2. This result can be attributed to the flexibility of the PEA chains along with presence of branches in the blend. Consequently, the free volume between the polymer chains is increased and the mobility of the polymeric chains is enhanced. Moreover, although PES is of hydrophobic character while PEA is a hydrophilic polymer with a large number of OH end groups, the obtained blends were compatible and no phase separation occurred and only one *T*_g_ was assigned.

**Table 2:** Properties of membranes with different blending ratios

Membrane	Water uptake (%)		Temp. at 10% wt loss (°C)	*T*_g_ (°C)
	Dist. water	Saline water		
M1	293	289	328	199.9
M2	349	319	284	165
M3	419	340	284	122.1

#### 3.3.3 Thermo-gravimetric analysis (TGA)

**Fig. 4:**
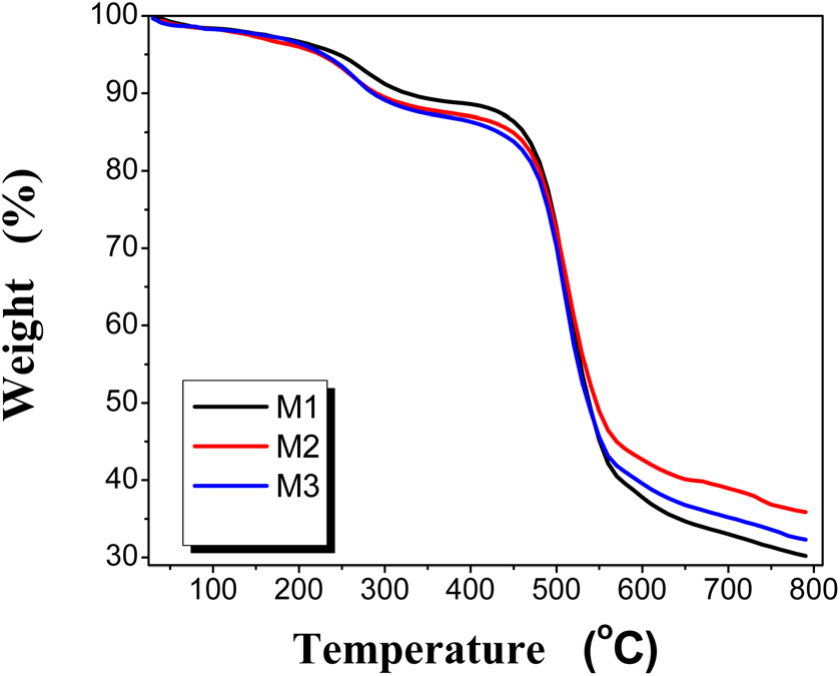
Thermo-gravimetric analysis (TGA) of the PEA/PES membranes.

Thermal stability of the PEA/PES was determined and the obtained thermograms are presented in Fig. 4 and the values of 10% weight loss are listed in Table 2. It can be observed that the membranes are thermally stable up to 250 °C. There is a gradual degradation starting at 220 °C due to the loss of residual solvent (NMP). However, one may observe that the decomposition temperature of M1 starts at 320 °C, losing 10% of its weight, and the temperature of 10 wt% loss is decreased in the cases of M2 and M3, which confirms the degradation of PEA. The second degradation step is considered for the polyether sulphone backbone. Therefore, it can be realized that blend membranes are thermally stable up to 250 °C. This type of behavior may be due to the stronger interaction between the two polymers and the high homogeneity of the blend.

#### 3.3.4 Water uptake

The prepared PEA/PES membranes are immersed in pure water and saline water to evaluate their water uptake. The ratio of water contents was determined for the three different membranes (Table 2). It was found that the membrane water uptake in pure water is higher than in saline water, and the water uptake is increased by increasing the PEA content in the blend. This result is related to the hydrophilicity and porosity of the membrane surface. It is believed that the presence of PEA in the blend introduced more pores to the membrane surface. The tendency of the prepared PEA/PES membranes for water uptake makes it challenging to use them in water purification.

#### 3.3.5 Mechanical property

To evaluate the impact of PEA/PES blend ratio on the mechanical behaviour of membranes, mechanical testing was carried out. The characteristic parameters were recorded at ambient temperature and the values are gathered in Table 3: Young modulus is determined from the initial slope, which is related to material elasticity. The results revealed that elongation at break of the PEA/PES membranes decreased with increasing PEA content in PEA/PES membranes, which can be attributed to a decrease in membrane's resistance to fracture. Besides, samples M1 and M2 showed higher values of Young's modulus and can be considered stiff. Increasing the amount of PEA in the membrane M3 led to a membrane of lower stiffness as indicated by lower values of elongation at break and Young's modulus.

**Table 3:** Mechanical properties of PEA/PES membranes with different blending ratios

Membrane	Young's modulus (MPa)	Tensile strength (MPa)	Elongation at break (%)
M1	73	2.98	5.69
M2	74	0.908	3.22
M3	35	0.764	1.62

#### 3.3.6 Contact angle

Investigation of the surface properties of polymers is of considerable interest for the prediction of their adhesion and wetting properties and surface polarity. The investigation of the membrane surface was carried out by measuring the contact angle using the captive air bubble method. The advantage of this method is that the measurements are done while the membrane is wet, which represents the normal condition for the membrane. Table 4 summarizes the mean values of the advancing and receding contact angles of the air bubble. The values of the contact angle for the PEA/PES blend membranes are lower compared to those of the pure polyethersulphone (PES) membranes. Moreover, the surface of the blend membranes is more hydrophilic than that of the reference PES. The low hysteresis also reflects the homogeneity of the blend. Annealed samples are more hydrophilic than wet samples. M2 is more hydrophilic than M3 since the amount of PEA in it is higher than that in M3 (as confirmed by experiments and calculations).

**Table 4:** Values of advancing angle (*θ*_a_) and receding angle (*θ*_r_) for prepared membranes

Sample	*θ*_a_	*θ*_r_
PES 15	55	33
M3_RT	58	35
M3 (80C)	38	34
PES 18	70	38
M2_RT	36	29
M2 (80C)	28	21

#### 3.3.7 SEM

**Fig. 5:**
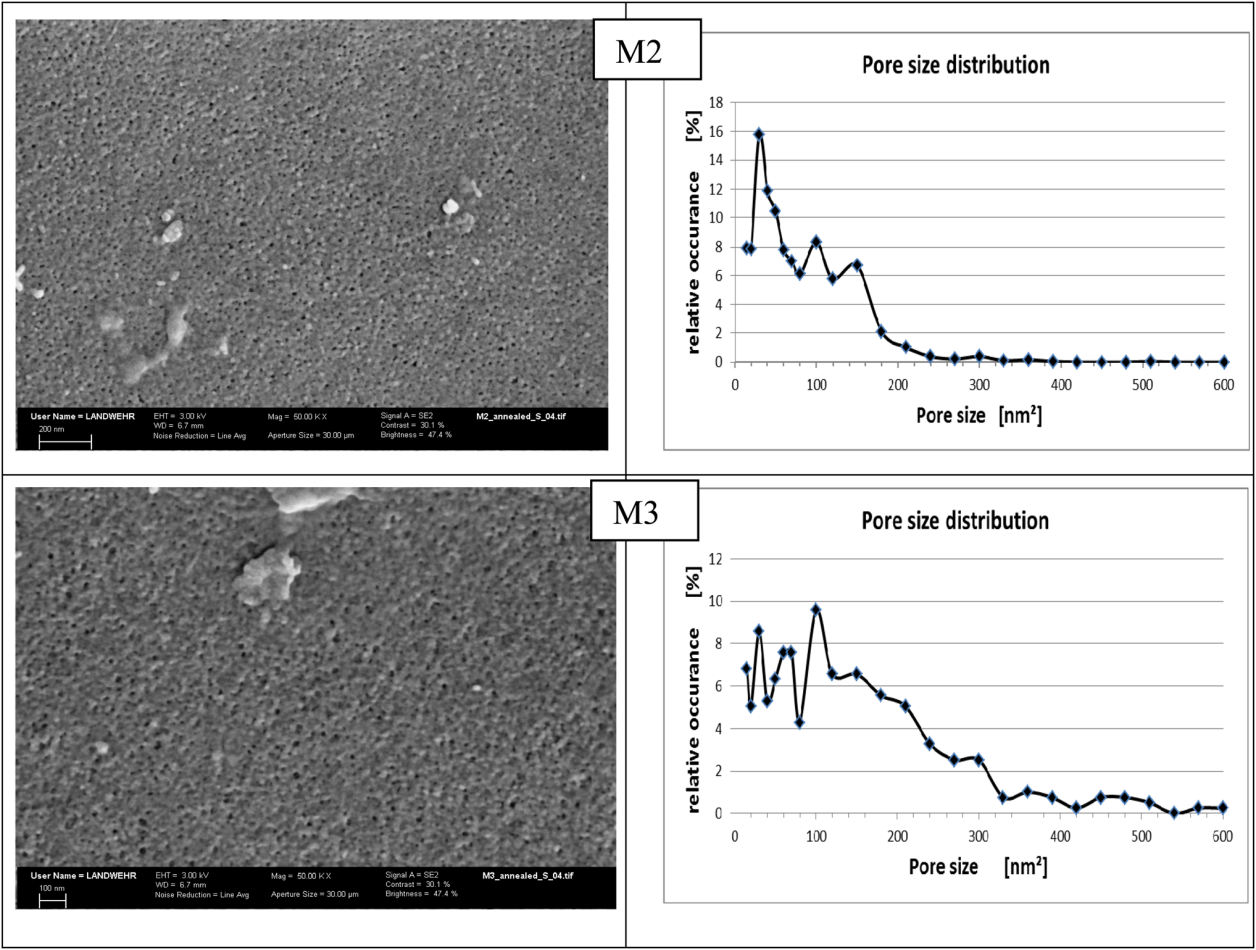
SEM images and pore size distribution for M2 and M3 membranes annealed at 100 °C (*X* = 50 000).

**Fig. 6:**
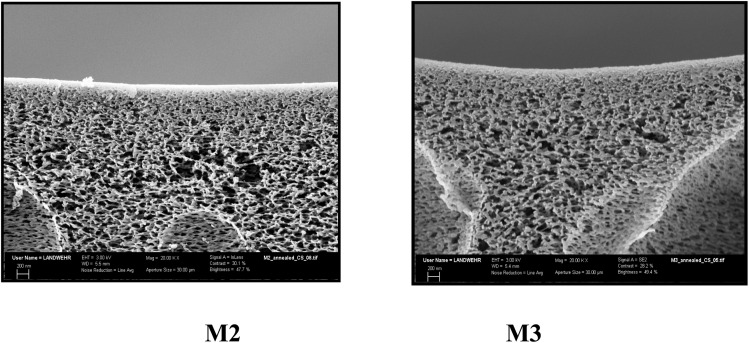
Cross section of M2 and M3 after annealing at 100 °C (*X* = 20 000).

The surface morphology of the prepared membranes M2 and M3 is studied by SEM, shown in Fig. 5, illustrating a rough porous membrane. Both membranes were annealed at 100 °C before testing; the reason for annealing is to eliminate tension in the membrane tissue after phase inversion through this relaxation process.^38^ Moreover, annealing creates changes in the membrane surface morphology and the active layer in depth and fixation of the membrane structure. It was found that membrane M2 has a more uniform pore size distribution as depicted in the curve obtained by processing the SEM image using the program Image J (Fig. 5). On the other hand, membrane M3 showed less homogeneity and larger pore size distribution as can be noticed in the graph on the right side although both membranes were annealed at the same temperature. Moreover, the size of the pores is larger in the case of M3 than that in M2. These results confirm that increasing the amount of HP–PEA in the membrane blend is responsible for the changes in the porosity and pore size. By exploring the morphology of the area underneath, it can be seen that both membranes have a similar cross-sectional morphology, exhibiting a spongy shape with a macrovoid structure (Fig. 6). These observations indicate that the effect of the presence of the hyperbranched polymer in the membrane blend is more pronounced in the membrane's surface porosity than under the active layer in the membrane tissue. This similarity in the membrane microstructure can be attributed to the high compatibility and homogeneity of the two polymer systems despite the known hydrophilicity of the HP–PEA compared to the rigid structure of PES.

### 3.4 Membrane performance

**Fig. 7:**
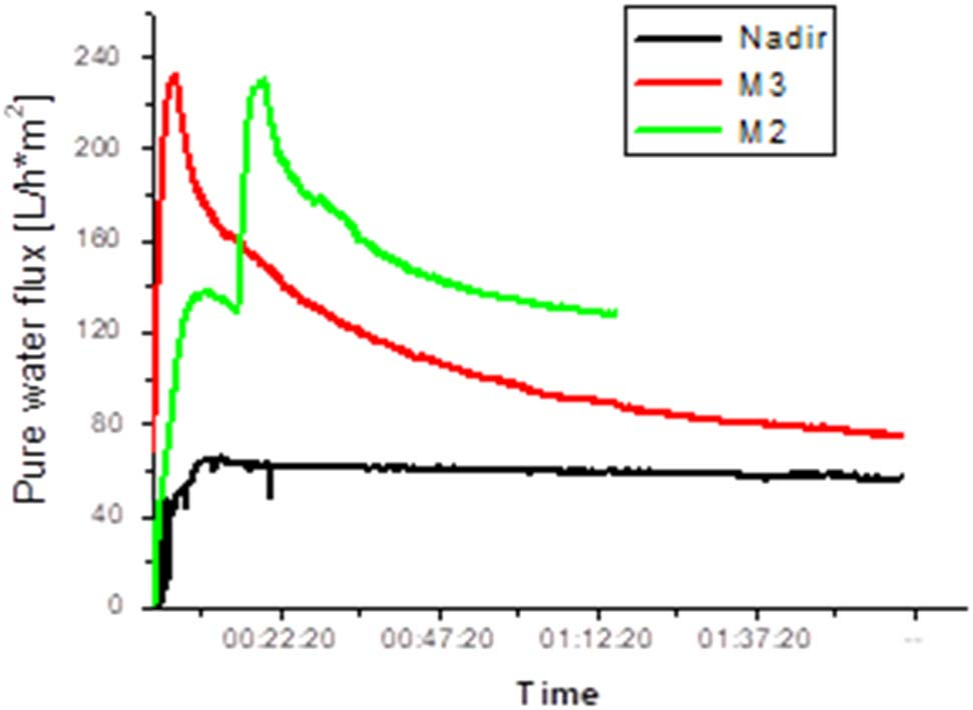
Variations in pure water flux for different membranes at constant pressure (4 bar).

The membrane performance tests in pure water at constant pressure (4 bar) were carried out for the prepared membranes in comparison with the pure PES analogs and a commercial NF membrane (Nadir). The performance data were taken after the membrane compaction stage for 2 hours. Generally speaking, it is well known that several factors including surface pore size, cross-section morphology, skin-layer thickness, and surface hydrophilicity together determine the permeation flux of the membrane. The blend membranes showed higher pure water flux than the commercial membrane as can be seen in Fig. 7. Membrane M2 exhibits a water flux of about 125 L h^−1^ m^−2^, which is double the value found for membranes prepared from pure PES (PES 15 and PES 18). On the other hand, the commercial membrane showed a pure water flux of 50 L h^−1^ m^−2^. It is obvious that the presence of a large number of pores on the blend membrane skin is the reason for the high water flux. Although all the tested membranes were annealed at the same temperature, M2 and M3 preserved their porosity unlike the pure PES membranes, which acquired a dense surface after annealing.

#### 3.4.1 Membrane performance for heavy metal removal

It is well known that several factors including surface pore size, cross-section morphology, skin-layer thickness, and hydrophilicity determine the permeation flux of the membrane together. The prepared membranes are examined for the removal of heavy metals such as Pb(NO_3_)_2_ and CuSO_4_. The concentration of Pb(NO_3_)_2_ solution is 200 ppm, while for the CuSO_4_ solution two different concentrations are tested which are 200 and 400 ppm. Table 5 shows the permeate flux, membrane permeability, and membrane rejection for different membranes.

**Table 5:** Pure water flux and salt removal for various membrane types

Sample	Annealing temperature	Salt type/concentration ppm	Permeate flux [kg h^−1^ m^−2^]	Membrane permeability [kg h^−1^ m^−2^ bar^−1^]	Rejection (%)
M2	Dry@r.t.	Pb(NO_3_)_2_/200 ppm	1581.59	399	1.9
M2	100	CuSO_4_/200 ppm	125.7	31.4	11
M2	100	CuSO_4_/400 ppm	70	17.5	37.5
M2	100	Pb(NO_3_)_2_/200 ppm	97.65	24.4	85.5
M2	120	CuSO_4_/200 ppm	101.74	25.4	41
PES 18	100	Pb(NO_3_)_2_/200 ppm	49	12.5	47.72
M3	100	CuSO_4_/200 ppm	63.7	15.9	43
M3	100	CuSO_4_/400 ppm	45.3	11.3	41
M3	100	Pb(NO_3_)_2_/200 ppm	56.96	14.2	81
PES 15	100	Pb(NO_3_)_2_/200 ppm	55	13	41.7

**Fig. 8:**
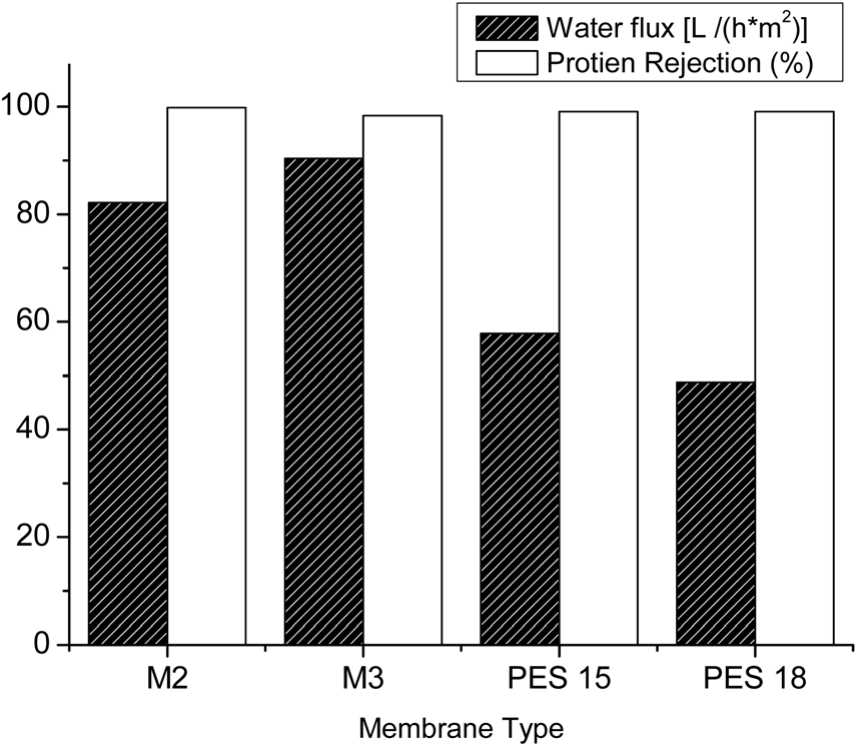
Membrane recovery and pure water flux for blend and pure PES membranes.

It was found that the blend membranes have high salt rejection compared to pure PES with a good permeate flux. M3 membrane has 81% rejection of Pb (NO_3_)_2_ and 41% for CuSO_4_. There is no big difference in using higher concentrations of heavy metal salt solution. Similarly, M2 rejected 85% of Pb (NO_3_)_2_ with good water flux compared to the PES membrane. It was found from these results that the separation depends on the heavy metal type and also its particle size (Fig. 8).

#### 3.4.2 Protein adsorption: protein–bovine serum albumin (BSA)

**Fig. 9:**
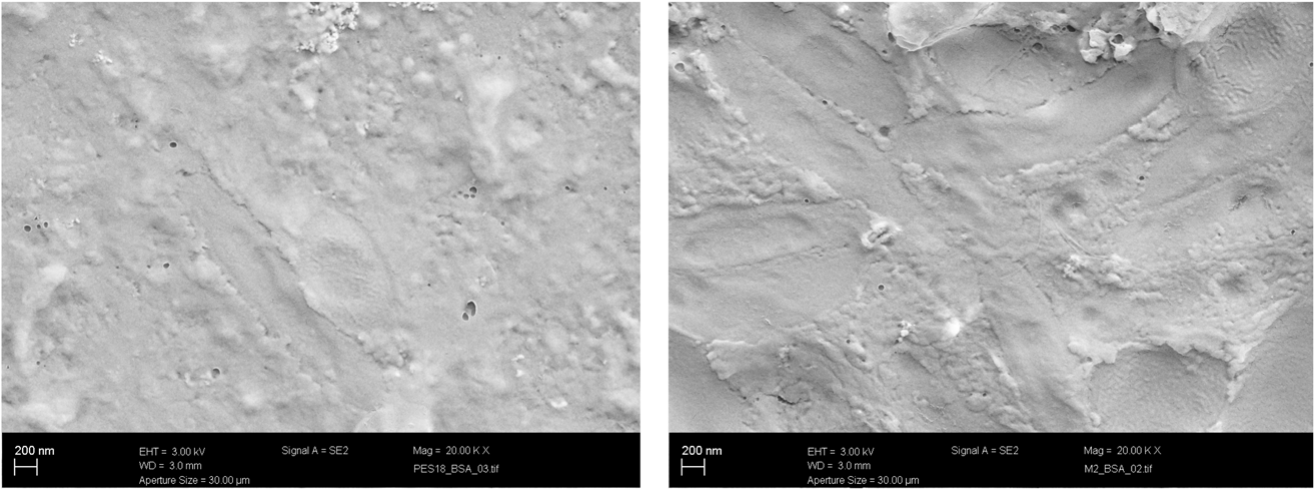
SEM micrographs for the M2 (left) and PES18 (right) membranes with the surface covered with BSA (*X* = 20 K).

The effect of blending PES with hyperbranched polyester-amide on the removal of protein–bovine serum albumin (BSA) was examined. The presence of PEA has led to reduced pores and protein rejection up to 99.5%. The protein rejection test was carried out in three steps; the first one was testing the permeability of pure water for 2 hours until membrane performance stabilization. The second step was testing the protein solution, while membrane recovery was tested once more using pure water in the third. Fig. 9 shows the membrane recovery and pure water flux. All tested membranes showed protein rejection not less than 99.5% and in general, the pure water flux is reduced due to the presence of a thick layer of protein on the membrane surface. Nevertheless, M2 and M3 have a water flux of 82 L h^−1^ m^−2^ and 90 L h^−1^ m^−2^, respectively, despite the presence of a thick layer of separated protein, which can be attributed to the larger pore size on the membrane surface.

## Conclusion

PEA/PES mixed matrix nanofiltration membranes were successfully prepared *via* the phase inversion method. The obtained membranes were characterized using FTIR and TGA and their morphology was investigated by SEM. Membranes with similar surfaces and cross-sections with a highly porous morphology could be produced. The mechanical behavior of PEA/PES recorded at ambient temperature revealed that the Young modulus, elongation at break and tensile strength of the PEA/PES membranes decreased with increasing the PEA content in PEA/PES membranes, which led to a decrease in the elasticity. Thermal analysis investigation showed that blending of PEA with PES led to an increase in the decomposition temperature up to 250 °C. This type of behavior may be due to the stronger interaction between the two polymers in the blend through electrostatic bonds. The values of the contact angle for the PEA/PES blend membranes are lower compared to those of the pure polyethersulphone (PES) membranes. Moreover, the surface of the blend membranes is more hydrophilic than that of the PES membrane. The newly prepared PEA/PES NF membranes exhibited high salt rejection compared to pure PES with a good permeate flux. Moreover, the presence of PEA has led to reduced pores and protein rejection up to 99.5%. The novel blend membranes proved themselves as NF-type membranes with better performance and suitable for water treatment compared to traditional PES membranes.

## Data availability

All data generated or analyzed during this study are included in this article.

## Conflicts of interest

There are no conflicts to declare.
